# NOVEL METABOLOMICS MARKERS PREDICT 18-MONTH DECLINE IN HAND GRIP STRENGTH IN COMMUNITY-DWELLING OLDER ADULTS

**DOI:** 10.1093/geroni/igz038.216

**Published:** 2019-11-08

**Authors:** Ted Kheng Siang Ng, Jean-Paul Kovalik, Jianhong Ching, Angelique Chan, David Matchar

**Affiliations:** 1 DUKE-National University of Singapore, Singapore, Singapore; 2 Duke-NUS Medical School, Singapore, Singapore; 3 Centre for Ageing Research and Education, Duke-NUS Medical School, Singapore, Singapore; 4 Duke-NUS Medical School, Singapore, Singapore, Singapore

## Abstract

Sarcopenia that accompanies aging necessitates early detection tools, ideally before the presentation of clinically evident symptoms. The acylcarnitines (ACs) are a class of metabolites generated by cellular fuel metabolism and their predictive utility in declining muscle strength in community-dwelling older adults is unknown. We aim to examine whether baseline acylcarnitines levels can predict changes in hand grip strength over 18 months in 121 community-dwelling older adults. We measured ACs by targeted plasma metabolomics profiling. We then performed a biologically-relevant classification of these markers. Hand grip strength was measured using a Smedley spring-type dynamometer. Multivariate linear regressions were performed to examine if: 1) there was an association between ACs and hand grip strength at baseline and 2) baseline ACs could significantly predict changes in hand grip strength over an 18-month period. At baseline, AC levels were not significantly associated with hand grip strength. We found an inverse association between baseline short-chain carboxyl and dihydroxl acylcarnitines (AC-DC/-OH) levels and 18-month changes in hand grip strength (p=0.047, β=-0.548, 95% CI=-1.088 to -0.008). Notably, a specific AC-DC/-OH species, C4-DC/C6-OH, accounts for the majority of the variance. The mean difference between Malay and Chinese ethnicity is 2.28kg (p=0.042, β=2.275, 95% CI=0.084 to 4.466). These findings suggest an association between metabolic markers and deterioration in hand grip strength. These results suggest that perturbations in fuel metabolism are detectable way before the emergence of clinically evident sarcopenia and frailty. The use of AC-DC/-OH panel as antecedent biomarkers may enable clinicians to risk stratify patients in the future.

